# Classification of Cortical Neurons by Spike Shape and the Identification of Pyramidal Neurons

**DOI:** 10.1093/cercor/bhab147

**Published:** 2021-06-12

**Authors:** Roger N Lemon, Stuart N Baker, Alexander Kraskov

**Affiliations:** Department of Clinical and Movement Neurosciences, UCL Queen Square Institute of Neurology, London WC1N 3BG, UK; Biosciences Institute, Newcastle University, Newcastle upon Tyne NE2 4HH, UK; Biosciences Institute, Newcastle University, Newcastle upon Tyne NE2 4HH, UK

**Keywords:** antidromic, cell types classification, interneurons, pyramidal, spike shape

## Abstract

Many investigators who make extracellular recordings from populations of cortical neurons are now using spike shape parameters, and particularly spike duration, as a means of classifying different neuronal sub-types. Because of the nature of the experimental approach, particularly that involving nonhuman primates, it is very difficult to validate directly which spike characteristics belong to particular types of pyramidal neurons and interneurons, as defined by modern histological approaches. This commentary looks at the way antidromic identification of pyramidal cells projecting to different targets, and in particular, pyramidal tract neurons (PTN), can inform the utility of spike width classification. Spike duration may provide clues to a diversity of function across the pyramidal cell population, and also highlights important differences that exist across species. Our studies suggest that further electrophysiological and optogenetic approaches are needed to validate spike duration as a means of cell classification and to relate this to well-established histological differences in neocortical cell types.

## Introduction

It is now widely recognized that understanding the operation of cortical circuits requires not only careful documentation of neuronal activity but also better identification of the neuronal cell types exhibiting that activity (Tremblay et al. 2016; Zeng and Sanes 2017). Technical advances have allowed better and better insights into the activity of large populations of cortical neurons. These approaches are being actively employed in a variety of animal models, including mice, rats, and macaque monkeys, with extracellular recordings from probes carrying multiple contacts (Barthó et al. 2004; Buzsáki et al. 2015; Michon et al. 2016; Jun et al. 2017; Angotzi et al. 2019).

Identifying the neuronal cell types involved has advanced more slowly. The classical approach of in vivo recording from an individual neuron, and then labeling the neuron and reconstructing it from the histology of the harvested tissue (Deschênes et al. 1979; Ghosh and Porter 1988; Pinault 1996) is not practical when recordings are being made over a long time period in an awake animal and from multiple neurons.

One of the most enduring ideas is that extracellular spike shape might help to distinguish different classes of neuron recorded in this type of study. Early investigators, recording in the awake monkey, first suggested that interneurons (stellate or Golgi type II), with high spontaneous firing rates, had “thin” action potentials of short duration and could be distinguished from putative pyramidal cells with broader spikes and a lower, regular spiking pattern of discharge (Mountcastle et al. 1969). These differences were subsequently confirmed by detailed electrophysiological investigations in rodent neocortex (Simons 1978; Connors et al. 1982; McCormick et al. 1985; Barthó et al. 2004; Contreras 2004). Other early investigations in rabbit neocortex used the antidromic response of pyramidal neurons with axons in the corpus callosum to distinguish them from other types of neuron, including interneurons (Swadlow 1989, 1990, 1991, 1994).

Spike-width characteristics were subsequently used by a number of investigators as a means of distinguishing interneurons from pyramidal neurons in extracellular recordings from the cortex of awake macaques (Merchant et al. 2008; Johnston et al. 2009; Kaufman et al. 2010; Ardid et al. 2015; Trainito et al. 2019). In these studies, the duration of the spike is usually measured from its negative trough to the following positive peak, reflecting the repolarization of the neuron, although other, closely correlated, measures of spike duration have also been used (e.g., in Swadlow’s work, the initial positive component is included in spike duration measurement). It is also important to note that spike duration measures are affected by the recording parameters such as the filter settings. Higher values for the high-pass filter cutoff frequency lead to the narrower spike duration measurements and the widest spikes are the most affected (see fig. 2 in Vigneswaran et al. 2011 for a detailed analysis of this issue).

Recent work has used a number of different spike waveform and discharge parameters to identify different classes of neuron recordings from prefrontal and posterior parietal cortex; these classes were then shown to differ in terms of their response dynamics and information coding (Ardid et al. 2015; Trainito et al. 2019). The classes with the briefer spikes were considered to be putative interneurons; those with the broader spikes as putative pyramidal cells.

Despite the increasingly widespread application of this approach (see e.g., Trainito et al. 2019), it is also recognized that misclassification of interneurons and pyramidal neurons might arise because, simply put, not all interneurons have “thin” spikes and not all pyramidal neurons have broad ones, and therefore classification based solely on spike duration may be problematic (see Swadlow 1994). Direct evidence of the latter, from recordings in the awake monkey, was provided by Vigneswaran et al. (2011), who showed that in both motor and premotor cortex there was a substantial proportion of layer V cells, positively identified as pyramidal neurons, that exhibited “thin” spikes.

### Pyramidal Tract Neurons (PTNs) in the Awake Monkey

PTNs arise from layer V: they are all pyramidal neurons that project their axons via the pyramidal tract in the medulla into the spinal cord. In the macaque, there are estimated to be more than 500 000 PTNs which arise not only from M1, but also from a number of different frontal and parietal cortical areas (Dum and Strick 1991; Porter and Lemon 1993; Firmin et al. 2014). They represent a functionally important part of the total corticofugal output from layer V. In the macaque, the axons of PTNs exhibit an almost 100 fold range in fiber diameter (Firmin et al. 2014), and there is growing evidence that they are involved in a wide range of functions, by no means restricted to motor control (Lemon 2008, 2019). Different subpopulations of pyramidal neurons can also be distinguished on the basis of their discharge characteristics and responses (Ardid et al. 2015; Trainito et al. 2019).

**Figure 1:**
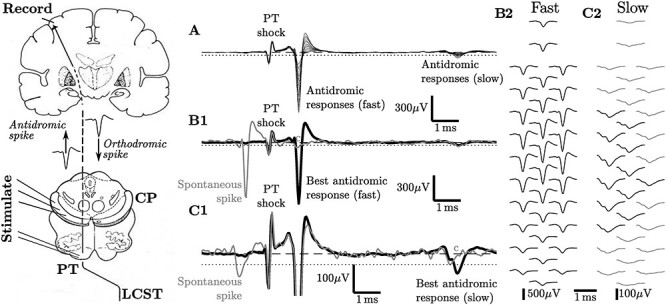
**Macaque “fast” and “slow” M1 PTNs recorded with a polyprobe electrode.** The diagram on the left shows the method for antidromic identification of pyramidal neurons with subcortical projections (e.g., corticospinal neurons with axons in the pyramidal tract (PTNs) and lateral corticospinal tract (LCST) or other corticofugal neurons with axons passing through the cerebral peduncle (CP). These neurons, recorded in motor cortex (M1), show an antidromic response to stimulation of the medullary pyramidal tract (PT) or cerebral peduncle (CP) delivered via fine, implanted electrodes. The action potential generated by stimulation is shown traveling antidromically toward the cell body in the cortex. If a projecting neuron fires a spontaneous spike shortly before the stimulus is delivered there is collision in the axon between this spontaneous spike, traveling orthodromically and the antidromic spike, and the antidromic response recorded at the cortex is extinguished (see Lipski 1981). The records on the right were obtained from a 32-channel polyprobe electrode advanced into M1 cortex of an anesthetized adult macaque until the tip was at a depth of 1.1 mm. (**A**) shows superimposed averages of records obtained from all 32 contacts (53 sweeps per average) in response to a single shock (72 μA) delivered to the ipsilateral pyramidal tract (“PT shock”). The earliest response represents the early spike from a “fast” PTN (antidromic latency 1 ms), while the later responses at around 7 ms are spikes from two slow PTNs (antidromic latencies 6.6 and 7.1 ms, respectively). Averages of the channel yielding the largest responses are shown in **B1** for the fast PTN and **C1** for the slow PTNs. Note the different scaling in amplitude; the fast PTN was much larger. The light gray single sweeps show examples of collision of the antidromic spike by an orthodromic spike occurring just before the PT shock. In the case of the slow PTNs in **C1**, the PTN with the slightly longer ADL (and larger spike) fired just before the PT shock, and the smaller, earlier PTN was still present in the response. The trough-to-peak duration of the fast PTN was 240 μs, whereas for the PTN showing collision in **C1** it was 760 μs. **B2** and **C2** show the averaged antidromic responses for the fast and slow PTNs, respectively. Note that the spike from the fast PTN (**B2**) was detected on all 32 contacts, although with a wide range of amplitudes. The slow PTN that shows collision in **C1** was only clearly detected in recordings from around 12 of the contacts (**C2**; black traces) and was too small to be discriminated on the others (gray traces). We used a 32 channel polyprobe (see Neuronexus catalog A1x32-Poly3-5 mm-25 s-177) with 15 μm contact diameter. Contacts were arranged in three columns separated by 18 μm and shifted by half intercontact distance against each other. Middle column contained 12 contacts and side columns had 10 contacts. Intercontact distance within each column was 25 μm, which gives 22 μm intercontact distance between nearby contact in different columns. The contacts extended a total distance of 275 μm vertically and 36 μm horizontally, as measured between centers of most distant contacts vertically and horizontally.

PTNs can be identified by their antidromic responses to stimulation of the pyramidal tract in the medulla (diagram on the left of Fig. 1). There are a number of well-established tests to verify the antidromic nature of the responses (Lipski 1981). These include demonstration of an invariant antidromic latency (ADL), as indicated by the superimposition of a large number of individual responses. An example from a so-called “fast” PTN, with a fast-conducting axon and short ADL is shown in Figure 1 B1 (variation of the spike shape across multiple contacts of the polyprobe is shown on Fig. 1 B2), and from a “slow” PTN with a slow-conducting axon and longer ADL in Figure 1 C1, C2. A second test is the collision of the antidromic spike when the stimulus is appropriately timed with respect to a spontaneous or orthodromic spike from the same unit (Fig. 1; see gray single traces in Fig. 1 B1 and C1). Other tests include demonstration of a sharp threshold and the capacity to follow a high-frequency train of stimuli.

**Figure 2:**
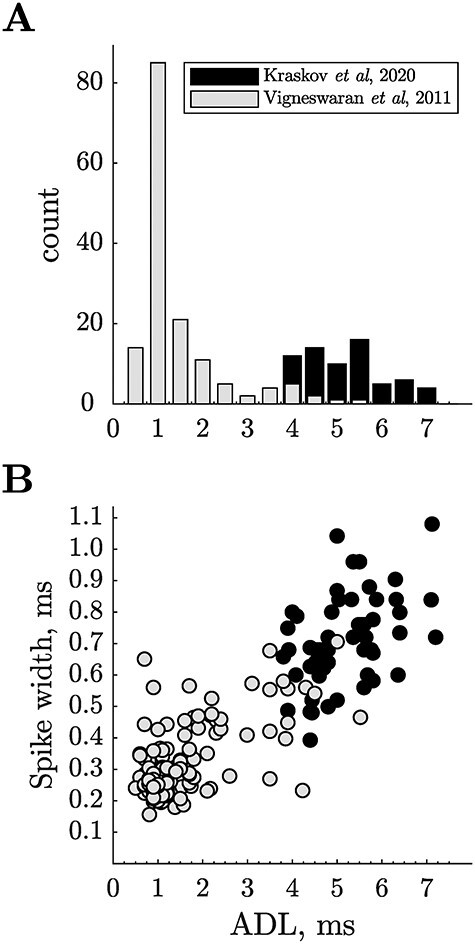
**Antidromic latencies and spike durations of “fast” and “slow” PTNs. A**. Comparison of antidromic latencies of 67 slow PTNs recorded from M1 in anesthetized macaques using polyprobe electrodes (Kraskov et al. 2020; black bars), with latencies of 151 M1 PTNs in awake macaques using single microelectrodes (Vigneswaran et al. 2011; light gray bars); most of these were fast PTNs. **B**. Trough-to-peak spike duration for slow PTNs (Kraskov et al; black circles) plotted against the ADL of each PTN. Data from the Vigneswaran study (mostly fast PTNs) are shown in gray circles. There was a significant positive correlation between ADL and spike duration for both PTN samples (c = 0.44, *P* < 0.001 and c = 0.63, *P* < 0.001 for slow and fast PTNs, respectively). Note that many of the fast PTNs had brief spikes (160–300 μs), while slow PTNs had spikes ranging from 400 up to 1100 μs. These macaque data confirm earlier studies in the cat (see fig. 6 in Takahashi 1965 and fig. 1B in Calvin and Sypert 1976).

The ADL is a measure of the conduction velocity of the PTN’s axon. The distribution of ADLs for a population of 151 PTNs recorded by Vigneswaran et al. (2011) in M1 of awake, behaving macaques is shown by the gray columns in Figure 2A. They found a significant positive correlation between antidromic latency and extracellular spike duration determined from the trough-to-peak measure (Fig. 2B, gray symbols), with the “thinnest” spikes coming from PTNs with the shortest latencies. These results bear out much earlier studies in the cat (see fig. 6 in Takahashi 1965 and fig. 1B in Calvin and Sypert 1976).

**Figure 3:**
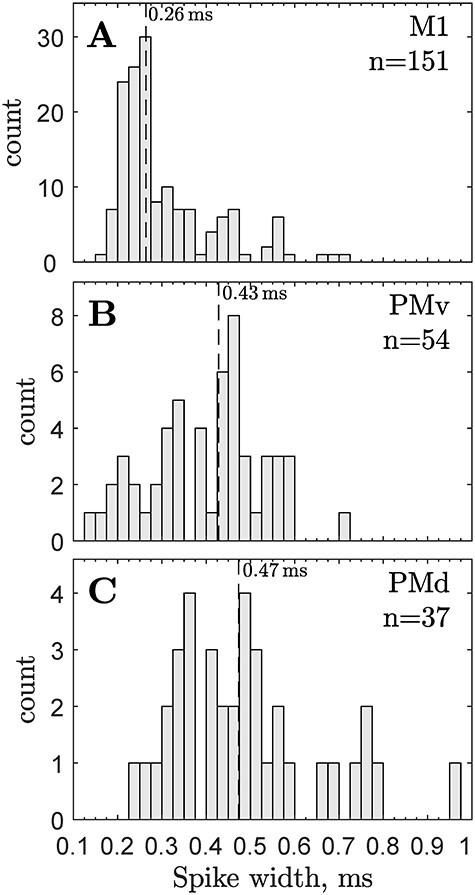
**Spike durations in pyramidal cells recorded in three different motor areas.** Distribution of trough-to-peak spike durations of neurons recorded in M1, ventral (PMv), or dorsal (PMd) premotor cortex and responding antidromically to stimulation of either the PT or the cerebral peduncle. Data for A and B from Vigneswaran et al. (2011) and for (C) from Lemon et al. (2012). PMv and M1 recordings made in awake macaques, PMd data in anesthetized macaques.

The detailed distribution of spike durations for the 151 M1 PTNs is shown in Figure 3A; the median spike duration was 260 μs, and the majority of M1 PTNs reported had spike durations of less than 350 μs. The briefest spikes were around 160 μs and came from PTNs with very short ADLs (<1 ms). This study was, like many others on M1 PTNs, dominated by recordings from fast PTNs (see Kraskov et al. 2019, 2020). PTNs with long ADLs (>5 ms) were very rare in their sample, but when present had some of the broadest spikes, around 500–700 μs in duration.

A recent study focused on the more slowly conducting PTNs (Kraskov et al. 2020), recorded using a polyprobe electrode under general anesthesia. In the macaque, they recorded 69 “slow” PTNs, with ADLs from 4 to 7 ms (Fig. 2A black columns). All these slow PTNs had broad spike durations, ranging from 400 to 1100 μs (black symbols in Fig. 2B). The two populations shown in Figure 2 may not be directly comparable, since general anesthesia may result in small changes in spike duration, but not to the extent that would blur the very big difference in spike duration for fast vs slow PTNs.

Taken together, these results suggest that PTNs in macaque M1 can have spikes with durations ranging all the way from “thin” (160–200 μs) right up to 1000 μs or more, values which span the entire range of spike durations reported in the literature to date. These spikes all come from one defined cell type, and therefore indicate that spike duration alone cannot be used to discriminate safely between putative interneurons (generally assumed to have brief spikes) and pyramidal neurons (generally assumed to have broad spikes).

### Other Potential Explanations of “Thin” Spikes in Macaque PTNs

Could there be an alternative interpretation that would question such a conclusion? For example, could these recordings come from axons rather than from the cell bodies of PTNs, since the former generally have brief extracellular spikes (Lemon 1984)?

This seems unlikely for a number of reasons: first, in our recordings, PTNs typically have large amplitude spikes and high stability, with many PTNs recorded for over an hour or more. Stability of recordings is a requirement not only for initial antidromic identification and the online collision test, but also to allow continuous recording of the PTN for several hundred trials of the monkey’s trained task. In contrast, recordings from axons are generally small, and only transiently present (Robbins et al. 2013; Godlove et al. 2014).

Furthermore, fast PTNs can be recorded over a considerable intracortical distance of at least several hundred μm. With the polyprobe electrode, the example of the fast PTN in Figure 1 could be detected on every one of 32 different contacts on the same probe, as shown in Figure 1 B2—a spatial extent of at least 275 μm.

The action potentials recorded from slow PTNs are generally smaller in amplitude than spikes from fast PTNs, and are generally detected on a smaller number of contacts (Fig. 1A, C1, C2; Kraskov et al. 2020). Modeling of data such as that shown in Figure 1 B2 and C2 indicates that these amplitude differences are real and cannot be explained by the relative distance of the PTN cell bodies from the probe contacts. There is a well-known recording bias toward large PTNs (Towe and Harding 1970) and the smaller spikes generated by slow PTNs probably explain why recordings from them are generally missing from published studies (see Kraskov et al. 2019, 2020). Clearly, a “thin” spike is not necessarily associated with neurons having long axons, since both fast PTNs with “thin” spikes and slow PTNs with broad spikes project axons at least as far as the lower cervical cord (Kraskov et al. 2020).

### Is Motor Cortex the Only Cortical Area where Potential Miscategorization Using Spike Shape Might Occur?

It could be assumed that because M1 is the main source of fast PTNs with “thin” spikes, miscategorization would not be a problem in recordings from other cortical areas. This still remains to be determined. For the time being, it is clear than in addition to M1, pyramidal neurons with “thin” spikes can also be found in secondary motor areas such as ventral premotor cortex (PMv; Vigneswaran et al. 2011; Fig. 3B) and dorsal premotor cortex (PMd; Lemon et al. 2012; Fig. 3C). Some of the PTNs in PMv exhibited very brief spikes (<200 μs), although in general the population had broader spikes than from M1 PTNs. The median spike duration was 430 μs vs 260 μs in M1. Even the small sample of 37 pyramidal cells from PMd shown in Figure 3C suggests it also contains pyramidal cells which can exhibit relatively brief spikes.

In an earlier study, Johnston et al. (2009) identified macaque corticotectal neurons in prefrontal cortex by antidromic stimulation of the superior colliculus. They found some of these pyramidal neurons to have relatively narrow spikes (briefest 287 μs, median 355 $\mu$s) although other unidentified neurons had much briefer spikes, and were considered to be putative interneurons.

In our view, this last study emphasizes why we need more studies of pyramidal neurons identified by antidromic stimulation of their projecting axons. These include PTNs across the broad cortical territory known to give rise to the PT (Dum and Strick 1991; Porter and Lemon 1993), including the supplementary motor area, cingulate motor areas and parietal areas such as S1 (see Vigneswaran et al. 2011) and SII. Other layer V corticofugal neurons giving rise to projections to the striatum, colliculus, midbrain, pontine nuclei, and brainstem (Kuypers 1981; Ugolini and Kuypers 1986; Turner and DeLong 2000) can be identified by antidromic stimulation of their cortical or subcortical targets (as in Johnston et al. 2009) or of the cerebral peduncle (see diagram on the left of Fig. 1; and Lemon et al. 2012). Pyramidal neurons with projections traveling in the corpus callosum can also be identified antidromically (Soteropoulos and Baker 2007).

### Is the Prevalence of Pyramidal Neurons with “Thin” Spikes Limited and Therefore Unlikely to Lead to Significant Errors of Categorization?

It might be argued that if PTNs with “thin” spikes represent only a small proportion of all pyramidal cells, there is no significant problem in attributing brief spikes to putative interneurons. Clearly it is a question of where the line is drawn. In one recent study of a number of temporal and prefrontal cortical areas, Torres-Gomez et al. (2020) found a bimodal distribution of spike widths, and they distinguished narrow from broad spiking neurons with a boundary between them close to a spike width of 300 μs. Vigneswaran et al. (2011) found that 64% of M1 PTNs (96/151) and 22% of PMv PTNs (12/54) had spike widths of <300 μs, so this suggests that the errors might be quite large.

At present we don’t know what percentage PTNs represent of all the pyramidal neurons in layer V, and in any case we first need to know whether or not other corticofugal pyramidal neurons exhibit brief spikes. There is also the question of the extent to which recording bias toward pyramidal neurons, with large cell bodies and open field structures vs interneurons with small cell bodies and closed structures, affects the overall picture (Kraskov et al. 2019, 2020). For the moment all these considerations should urge caution in attributing “thin” spikes exclusively to putative interneurons, with special caution when recording in layer V within macaque motor areas.

### The Importance of Species Differences

It was electrophysiological investigations in rodents that first suggested that “thin” spikes came from interneurons, while broad spikes emanated from pyramidal neurons (Connors et al. 1982; McCormick et al. 1985; Barthó et al. 2004; Contreras 2004). Recordings from antidromically identified pyramidal neurons with efferent projections and from suspected interneurons in awake rabbits suggested a similar tendency, although with a significant overlap in spike widths between the two populations (Swadlow 1989, 1990, 1991, 1994). In the cat, Takahashi (1965) and Calvin and Sypert (1976) showed that sensorimotor cortex “fast” PTNs had narrow spikes, and that there was a clear negative relationship between conduction velocity and spike duration.

Nevertheless, many recent studies in the awake macaque have used the rodent-based spike width criteria, having assumed that they can be applied to primates (Constantinidis and Goldman-Rakic 2002; Mitchell et al. 2007; Diester and Nieder 2008; Cohen et al. 2009; Johnston et al. 2009; Kaufman et al. 2010; Song and McPeek 2010; Ardid et al. 2015).

Important species-specific differences should not be ignored. For example, in macaques and other large primates, including humans, there is a population of large PTNs with fast-conducting axons (up to 80 m/s) which is completely lacking in the rodent, in which the fastest PTN axons conduct at around 18–20 m/s (see Lemon 2019). In the primate, this fast-conducting population is strongly implicated in the cortical control of fine movements of the hands and digits (Lawrence and Kuypers 1968; Lemon 2008, 2019; Quallo et al. 2012; Morecraft et al. 2013). Fiber systems in the brain that include some large, fast-conducting axons may be important in overcoming long conduction delays, as well as allowing for secure transmission of high-frequency discharges (Perge et al. 2012); these properties are no doubt important for the long corticospinal projections to cervical and lumbosacral cords in large primates, projections that are known to carry discharges with high instantaneous frequencies (Porter and Lemon 1993; Xu and Baker 2018). The brief spikes exhibited by large PTNs with fast-conducting axons are consistent with mechanisms for rapid repolarization essential for such high-frequency bursts of activity. We obviously need to know more about differences in discharge characteristics between rodent and primate PTNs.

It is well known that one of the determinants of fast repolarization and “thin” spikes in parvalbumin-expressing (PV+) cortical interneurons is the presence of the fast K+ channel Kv3.1b in their soma membranes (see Bean 2007). Macaque PV+ interneurons in M1 also express Kv3.1b (Soares et al. 2017).

**Figure 4:**
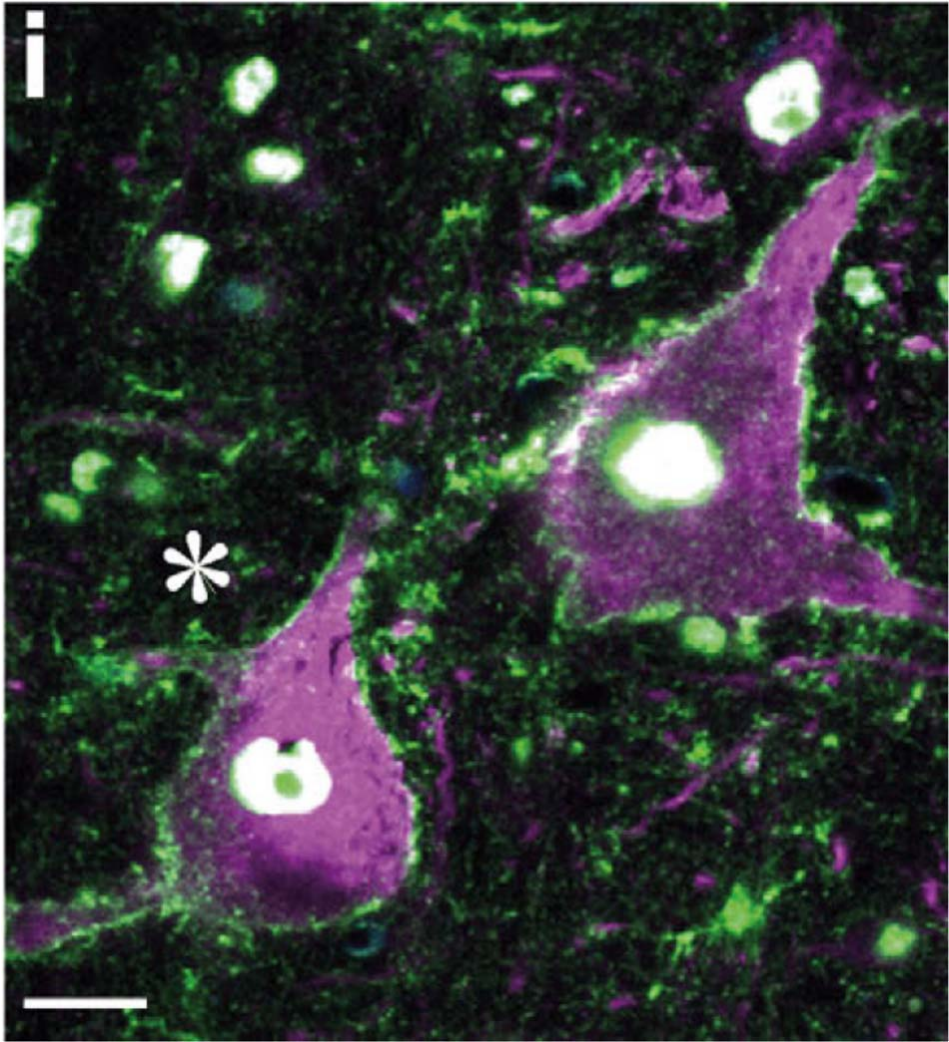
**Kv3.1b expression in the membranes of macaque M1 pyramidal neurons.** A section from M1 labeled with SMI32 (magenta) and Millipore antibody to Kv3.1b (green). The cell bodies of two pyramidal neurons, both positive for the neurofibrillary marker SMI32 can be seen, and in both cases the soma and proximal dendritic membrane expresses Kv3.1b (cf. Fig 4i, Soares et al. 2017). Scale bar 20 μm.

In contrast, pyramidal neurons in the rodent motor cortex lack Kv3.1b channels. However, in keeping with the observation of “thin” spikes in macaque PTNs, we published recent evidence that in macaque M1 cortex, Kv3.1b is also present in the soma-dendritic membrane of many layer V pyramids (Soares et al. 2017). These pyramidal neurons were identified both morphologically and by co-expression of the neurofilament marker SMI32, and included some of the largest pyramids, the so-called Betz cells, with heavy Kv3.1b labeling of the soma and proximal dendrites (Fig. 4). Interestingly, Kv3.1b expression in macaque pyramidal cells is not limited to M1, but has also been reported in V1 (Ichinohe et al. 2004; Constantinople et al. 2009).

### Evidence from Intracellular Recordings

There are relatively few reports in the in vivo macaque neurophysiological literature where investigators have actually identified the neuronal type from which recordings have been collected (Matsumura 1979; Hamada et al. 1981; Ghosh and Porter 1988). In what is admittedly a very small published sample, these cases include some PTNs with brief spikes (e.g., Matsumura 1979; see Fig. 3A).

There is of course a much larger literature on in vitro recordings from brain slices. Many of these data can now be referenced on the Allen database (https://celltypes.brain-map.org), where the intracellular recordings have been identified as coming from either spiny, sparsely spiny, or aspiny neurons, in slices from either mouse or human brains. This dataset shows a very clear contrast between spiny (pyramidal) neurons with generally broad spikes and aspiny (interneurons) with narrow spikes, and this is true for both mouse and human cells.

It is not surprising that the macaque PTN data summarized differ from the mouse layer V data, given that rodent pyramidal cells are almost exclusively characterized by broad spikes. It is also important to point out that the Allen database has relatively few layer V spiny (pyramidal) neurons from human cortex (71/2333) and, as far as we can determine, none at all from human motor cortical areas, tissue that is very rarely available for study.

There are some exceptions: for example, the database includes a spiny cell recorded from layer VI of middle temporal gyrus (ID 558211203) with an intracellular spike width at half amplitude of only 340 μs: this measure roughly corresponds to the trough-to-peak measure of spike width in extracellular recordings (Xu and Baker 2018). These investigators made paired intracellular and extracellular recordings from neurons in different layers of macaque M1 slices, and then filled the recorded neurons for later histological identification. But in general, just as in the Allen database, Xu and Baker (2018) report a striking absence of pyramidal neurons with brief spikes (trough-to-peak or width at half maximum <1000). This needs to be resolved; one possibility is that reflects the relative vulnerability of the largest neurons in in vitro preparations (Carp et al. 2008; Mitra and Brownstone 2012).

The Allen database also shows some interneurons with relatively broad spikes (e.g., aspiny cells recorded from layer V of the middle temporal gyrus (IDs 569 860 147 and 569 825 715) with spike widths of 920 and 780 μs), and this confirms a number of investigations looking at intracellular spike shape of morphologically identified interneurons in the macaque monkey prefrontal cortex (González-Burgos et al. 2005; Krimer et al. 2005; Zaitsev et al. 2009). These authors reported curved arbor cells and Martinotti cells with an intracellular spike width at half amplitude of 680 μs (Zaitsev et al. 2009). Another class of interneurons, double bouquet cells, have an even wider spike width of 740 μs. Interestingly these interneurons were all found to be PV negative.

## Conclusions

This commentary has emphasized that where electrophysiological identification has been possible through antidromic testing, the results suggest that spike shape alone cannot provide a reliable means of differentiating at least one sub-type of pyramidal cells, the PTNs, from interneurons. We are reiterating caution on this point, first made many years ago (Swadlow 1994) and repeated since (Vigneswaran et al. 2011), our concern being strengthened by new knowledge on the range of spike widths exhibited by macaque pyramidal cells and the striking differences between rodent and macaque in the expression of at least one membrane K+ channel that helps to determine spike width. The perils of misclassification are considered by some investigators to be limited (e.g., Trainito et al. 2019). However, given that pyramidal neurons with “thin” spikes have now been documented in more than one cortical area and in three different species (rabbit, cat, and macaque), greater caution is surely needed. In particular, the existence of PTNs with “thin” spikes in motor areas of macaque cortex should not be ignored, but rather should encourage investigation of PTNs in other cortical sensorimotor areas, and indeed of other layer V corticofugal neurons in wider regions. The use of correlation techniques for comparing the actions of pyramidal neurons with those of interneurons (Tamura et al. 2004; Merchant et al. 2008; Ren et al. 2020) should also be extended. Firing rate related measures should also be included into consideration (e.g., Zaaimi et al. 2018). All efforts to classify cell types using measures derived from extracellular recordings are important since it may potentially lead to the understanding of the computations performed by different classes in awake behaving animals. But we would like to emphasize that linking such classifications to anatomical, morphologically based classifications is not easy and requires caution. In the macaque, no doubt, better characterization of neuronal sub-types will be achieved using genetic dissection and optogenetic stimulation, exploiting the huge advances made in the mouse.

## Notes

We would like to thank all our lab members who helped to record and analyze identified pyramidal tract neurons. A.K. is supported by Wellcome Trust (02849/Z/13/Z) and BBSRC (BB/P006027/1). S.N.B. is supported by the Medical Research Council (MR/P012922/1, MR/P023967/1) and NIH (1R01NS119319-01). *Conflict of Interest*: None declared.
